# Metatropic Dysplasia of Nonlethal Variant in a Chinese Child – A Case Report

**DOI:** 10.1111/os.12546

**Published:** 2019-12-06

**Authors:** Michele A Tchio Tchoumba, Yan Bai, Runming Jin, Xianying Yu, Musa Male

**Affiliations:** ^1^ Pediatric Department, Union Hospital, Tongji Medical College Huazhong University of Science and Technology Wuhan China; ^2^ Pediatric Department Zhumadian City, The First People's Hospital Zhumadian China; ^3^ Department of Urology, Tongji Hospital, Tongji Medical College Huazhong University of Science and Technology Wuhan China

**Keywords:** Diagnosis, Metatropic dysplasia, Nonlethal metatropic dysplasia, Spondylometaphyseal dysplasia, Kozlowski type, Transient receptor potential vanilloid 4

## Abstract

Metatropic dysplasia (MD), is a rare skeletal dysplasia occurring predominantly in infants characterized by a distinctive long torso and short limbs; it is as a result of mutations in the TRPV4 gene. However, a clear distinction between various forms of skeletal dysplasias caused by the transient receptor potential vanilloid 4 (TRPV4) gene is difficult but could be achieved by a combination of gene sequencing, medical and radiological criteria. We hereby report a case of a 14‐month old girl who presented with an abnormal stature. The diagnosis of nonlethal MD was confirmed by X‐ray with dumbbell‐shaped long bones, platyspondyly, and delayed carpal ossification, as well as broadened pelvis with marginally widened ilia, epiphyseal plates, and slightly flattened acetabula. Furthermore, gene sequencing confirmed gene mutation on exon 15 of the TRPV4 gene with a heterozygous missense mutation (c.2396C > T), but no mutation was present in her parents. Our findings recorded metatropic dysplasia with the c.2396C > T mutation in the TRPV4 gene in China. This mutation caused changes in amino acid of TRPV4, which can induce growth retardation in children.

## Introduction

Metatropic dysplasia (MD) or changeable dysplasia has been characterized by progressive spinal changes observed from the neonatal period in childhood with a wide severity range both clinically and radiographically^1^, ^2^. MD is an autosomal dominant disease and one of the skeletal disorders caused by the loss of function of the transient receptor potential vanilloid (TRPV4) gene^3^. Usually, MD emerges in childhood when the child initially appears to have shorter limbs, a narrow chest, and long thorax but progressively changes with disease progression mainly due to kyphoscoliosis characterized by a shorter trunk^3^.

Dysplasia, regardless of the type, is generally diagnosed based on several parameters including radiological findings, physical examination, molecular and biological tests^4^. Clinical manifestation in infants include a marked forehead, shortened limbs, kyphoscoliosis, coccygeal tail, occasionally a square jaw, and peculiar facial features^1^, ^2^. Newborns carrying MD typically appear to have distinctively shaped pelvises, irregular calcanei and tali, delayed carpal ossification, and widened proximal and distal metaphases of femora resulting in a “dumbbell‐shaped bone”^1^.

In the current study, we reported a 14‐month old child with clinical and radiological features of MD with mutation of the TRPV4 gene. On the other hand, genetic analysis of each parent was normal.

## Case Report

A 14‐month old female child was brought to the Pediatric Department of Union Hospital by her parents because of short limbs. She was born with a healthy and uneventful birth history, as stated by her parents. At 3 months of age, the infant could sit up with help from her parents, and her head lift was normal. Communication and locomotive abilities appeared to be normal considering her then current age of 14 months.

**Figure 1:**
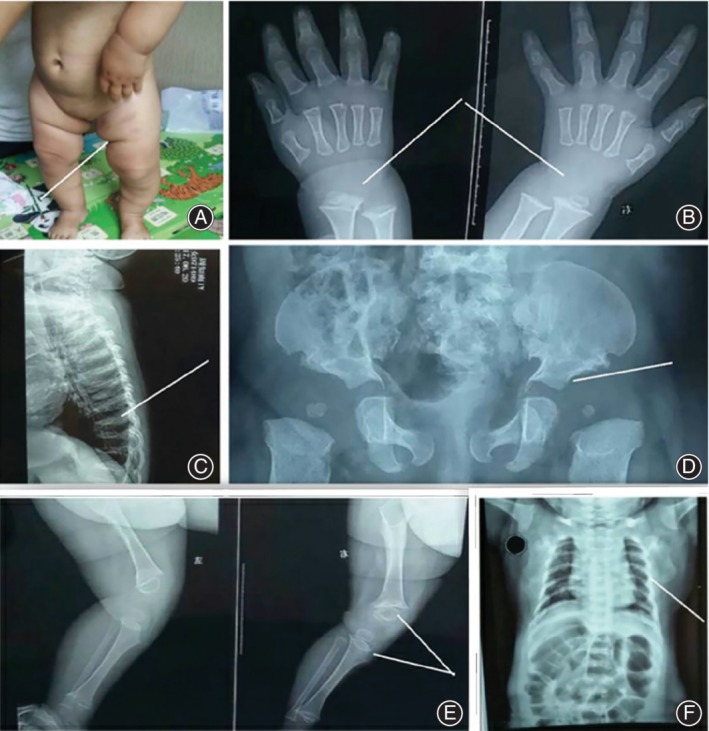
Physical and radiological changes in a Chinese infant with nonlethal MD. (A) Excessive skin creases on lower limbs. (B) Retardation of carpal ossification on anteroposterior (A/P) view (pointer). (C) Side view illustration of uneven margins and flattened vertebrae (platyspondyly). (D) Mildly uneven acetabula, slightly broad ilia, large epiphyseal plates are seen on pelvic A/P radiograph (pointer). (E) Flared metaphyses with reduced diaphysis of the lower extremities (pointer). (F) Illustrates a narrow thorax on A/P view.

Upon admission to our department, physical examination was performed, and this examination unveiled the existence of shorter limbs with redundant folds compared with her trunk. She was 73 cm tall (<50th percentile), 12 kg weight (98th percentile) with a head circumference of 46 cm (50th percentile). On radiological examination, the infant had a broad and irregular metaphyseal plate, with normal epiphyses. She also presented with a broad pelvis. The broadened pelvis, on the other hand, revealed slightly flattened acetabula, and marginally widened ilia and epiphyseal plates. Vertebral X‐ray of this infant later revealed flattened vertebrae (platyspondyly). A hand radiograph revealed apparent ossification delay considering her age **(**Fig. 1 **).**

**Figure 2:**
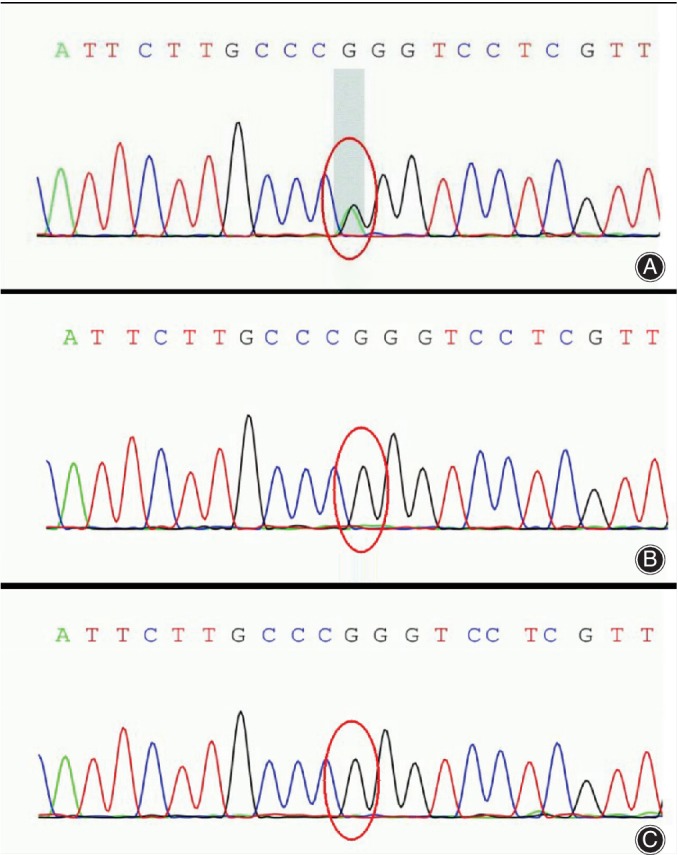
Illustration of TRPV4 mutation gene in a Chinese infant with nonlethal MD. (A) A heterozygous mutation of c.2396C > T at chr12:110222183 was showed *via* genome sequencing of the girl. (B) No mutation was found in her father. (C) No mutation was found in her mother.

Furthermore, gene sequencing confirmed gene mutation on exon 15 of the TRPV4 gene with a heterozygous missense mutation (c.2396C > T), but no mutation was present in her parents **(**Fig. 2 **).**

## Discussion

MD is a type of dysplasia characterized by dwarfism. It is classified among various forms of skeletal dysplasias caused by TRPV4 gene impairment which incorporates familial digital arthropathy‐brachydactyly, autosomal dominant brachyolmia, spondylometaphyseal dysplasia‐Kozlowski type (SMDK), spondyloepiphyseal dysplasia‐Maroteaux type (SEDM), parastremmatic dysplasia, and finally metatropic dysplasia^3^, ^5^. MD accounts for 5% of cases identified by the International Skeletal Dysplasia Registry^6^.

MD was first reported in 1893 as an atypical chondrodysplasia marked by short limbs, widened joints, and severe kyphoscoliosis^6^. Despite the appearance of several features associated with the disorder, some major phenotypes associated with this disorder are severe platyspondyly, shortened long bones, and dumbbell metaphyses^7^. MD appears to have a series of variants which include: (i) lethal form with distinct characteristics of semicircular iliac bones, shortened distance between successive pedicles, short diced ribs, underdeveloped chest, short voluminous pedicles, and long bones with extended mushroom metaphyses^8^; (ii) nonlethal dominant form characterized by progressive scoliosis, bone metaphyseal involvement, and delayed carpal ossification^1^; and (iii) nonlethal with autosomal recessive transmission^9^. Lethal forms are usually detected in the perinatal period through ultrasound and can be differentiated from the other forms *via* judge of phenotype severity and increment of mortality^10^. Nonlethal forms, on the other hand, present in childhood with short stature, failure of linear growth, or other physical abnormalities^4^.

Widened metaphyses leading to dumbbell appearance, small epiphyses, platyspondyly, and distinctive pelvic shape with flared ilia and horizontal acetabula are the radiological diagnostic criteria of MD^11^. However, the precise diagnosis is obstructed by the low efficiency of handling the complications and genetic counseling^4^. About 1/3 of patients diagnosed with lethal forms of MD died during childhood^12^. Mild MD or nonlethal MD in some cases may be recognized due to slight body disproportion, and kyphoscoliosis can be sighted until later in childhood after their short stature draws attention^3^. Generally, physical and mental development remains intact^12^.

Among all skeletal dysplasias associated with the TRPV4 gene mutation, SMDK has the most similar characteristics with MD. Spondylometaphyseal dysplasias (SMD) is a short stature disorder with an abnormality in vertebrae and metaphyses of tubular bones. SMDK is an autosomal dominant disease belonging to the class of SMD with particular radiological findings. It is characterized by wide proximal femoral epiphyseal plate, kyphoscoliosis, irregular metaphyses, delayed carpal ossification, flat acetabular roof, elongated vertebral bodies (overfaced pedicles), platyspondyly, and odontodysplasia^1^, ^10^. However, MD presents incredibly unique spinal, metaphyseal, and pelvic changes^9^. Initially, an anlage is at the origin of long bone formation whereby the bone elongates at both ends resulting in chondrocytes proliferation where it divides into two columns of pre‐hypertrophic and hypertrophic chondrocyte. Endochondral ossification is then initiated as blood vessels grow within the hypertrophic cartilage, which eventually gets resorbed and replaced by bone.

Consequently, the formation of growth plate occurs at both ends with a medullary cavity^13^. Improper alignment of proliferative chondrocytes with hypertrophic chondrocytes leads to the formation of nodules of various sizes and shapes in cartilage which is responsible for the enlargement of distal areas of long bones. This nodule occupies the region for growth plate leading to a flared and irregular metaphyseal plate^13^.

In previous reports, the nonlethal dominant form of MD is characterized by progressive scoliosis, bone metaphyseal involvement, and delayed carpal ossification, which is consistent with our findings in the infant. A remarkable radiological feature of MD, discriminating MD from other rhizomelia, is severe platyspondyly, which can be seen everywhere on the spine^14^. Changes in the spine in SMDK are very similar to nonlethal MD changes.

Furthermore, the view of the spine anteriorly (AP view) reveals overfaced pedicles and narrowing of interpedicular distance in the lumbar area and broad vertebral bodies extending beyond pedicles is seen in SMDK^2^, ^11^. Such features were absent in our patient despite overlapping spinal changes of SMDK and nonlethal MD. Besides, metaphyseal dysplasia in SMDK could be found until 4–5 years old^2^. Therefore, this finding is relevant to MD considering our patient is 14 months of age, and the X‐ray showed metaphyseal dysplasia.

Moreover, the long bones in SMDK will show mild flaring, hence the dumbbell appearance, which may be unnoticed, whereas dumbbell‐shaped bone in MD patients can be noticed, and this accentuated our diagnosis of nonlethal MD (Fig. 1E)^2^. Usually the thorax at birth in nonlethal MD appears narrow and slender while limbs appear shorter^3^. In SMDK, the trunk is short and broad compared to the narrow trunk exhibited in MD^2^.

TRPV4 is a gene which has several cellular functions and plays an essential role in intracellular calcium channel ion regulation, chondrocyte differentiation, and osteoclasts terminal differentiation. Therefore, its mutation will induce MD^11^, ^15^. TRPV4 gene is significantly expressed in several systems including nervous, respiratory, musculoskeletal, and urinary systems, in the vessel and even the eyes^3^. All the subtypes of MD have been shown subsequently to be the result of heterozygous mutations in TRPV4 2, 13. Most cases of mutations of MD occur as a result of novel mutations and, generally, can be seen in people with no recorded historical disorder in their family. Our patient's mutation was not detected in her biological parents.

Nevertheless, there are some exceptions in which the condition is inherited^6^, ^9^. However, previous studies revealed heterozygosity mutation involved in TRPV4 genes. Nucleotide substitution at c.G1781‐A in exon 11 was spotted in SMDK patients while nucleotide substitutions at c.C2396‐T and c.A991‐T in exon 15 were also identified in MD^1^, ^11^.

Moreover, more than 50 TRPV4 mutations have been documented, and exon 11 and 15 associated with SMDK and MD respectively are referred to a mutational hotspots^2^. In our case, the mutation was present in exon 15 on chromosome chr12‐110222183. A change occurred at c.2396C > T, which is a diagnostic mutation of MD.

The prognosis of MD varies, with the lethal form having the worst prognosis. Based on the reality of poor prognosis, kyphoscoliosis is a major progressive factor. The instability of the cervical spine can be the cause of quadriplegia or even sudden death. However, this clinical manifestation was not observed in our patient. Our patient seems to have quite a good prognosis. Finally, there is no effective treatment for MD. Palliative care is often offered^12^. It aims, above all, to combat the development of kyphoscoliosis by performing early vertebral arthrodesis. Beside causing dwarfism, MD can also induce osteopenia and fracture^3^. These fractures are referred to as insufficiency fractures mostly occurring in the metaphyseal plate of long bones^3^. In the case of damaged suboccipital hinge, surgical fixation of the atlas and the axis could be recommended. In the case of progressive kyphosis, bracing is the best choice and should be kept until maturity of skeleton or spinal fusion is attained. Impairment in extremities may necessitate osteotomies to improve its function. Early detection and diagnosis can be made in the perinatal period through ultrasound and has great importance for the management of complications. However, according to various studies, when children with MD reach adulthood, they are in worse health conditions than those without MD^8^. When the child grows, he or she might face situations of rejection, self‐dissatisfaction with their physical appearance, and isolation^12^. Even though a particular health care program is offered for such children while growing up to minimize complications, genetic counseling for the patients and their families can be helpful for early detection, decreasing complications, and ameliorates the patient's future lifestyle and self‐acceptance as the child grows. A multidisciplinary team of specialists is needed to help reduce the patient's handicap in society^8^, ^12^.

To conclude, our diagnosis of nonlethal MD is validated based on clinical manifestations, radiological findings, and TRPV4 gene mutation site identification. Although nonlethal MD and SMDK share very similar radiological findings, they can be discriminated by different TRPV4 gene mutation types. The early identification of nonlethal forms does not only reduce complications, but also ameliorates the patient's future lifestyle and self‐acceptance.

## Funding

None.

## Authors Contributions

MATT – drafted manuscript, YB – study design, XyY, ‐ collected data, RmJ ‐ analyzed data, MM and EK – reviewed and edited the manuscript.

## Ethics Approval and Consent to Participate

For ethical consideration, the patient's parents were informed about all data collection that might be used in this study in accordance with privacy rules. Nevertheless, approval was fully granted by the patient's parents.

## Consent of Publication

None.
